# Association between parents’ perceived social support and children’s psychological adjustment: a cross-sectional study

**DOI:** 10.1186/s12887-024-05235-7

**Published:** 2024-11-21

**Authors:** Rikuya Hosokawa, Toshiki Katsura

**Affiliations:** 1 Department of Human Health Sciences, Graduate School of Medicine, Kyoto University, 53 Shogoin-kawahara-cho, Sakyo-ku, Kyoto, 606-8507 Japan; 2 Faculty of Nursing, Meiji University of Integrative Medicine, Kyoto, 629-0392 Japan

**Keywords:** Social support, Child mental health, Parents’ perceived social support, Child psychological adjustment, Parent–child interactions

## Abstract

**Background:**

This study examined the relationship between parents’ perceived social support and their children’s psychological adjustment.

**Methods:**

This cross-sectional survey study was conducted in 52 kindergartens and 78 preschools in Nagoya, Aichi, a major metropolitan area in Japan. Parents of eighth-grade children aged 13–14 years (*N* = 1,195) completed a questionnaire. A total of 602 valid responses were received. To accurately assess the relationship between parents’ perceived social support and behavioral characteristics, respondents diagnosed with a developmental disability or who failed to answer the required questionnaire items were excluded from the analysis. Consequently, 536 (89.0%) of the 602 participants met the eligibility criteria.

**Results:**

The results indicated that the stronger the social support for parents, the lower the scores for externalizing and internalizing problems, and the higher the scores for prosociality. Conversely, insufficient social support may pose a risk to parental mental health and lead to suboptimal parenting practices. Issues in parental mental health adversely affect parenting, leading to fewer positive interactions with young children, increased rates of negative interactions and hostility, diminished communication, and delayed responses to children’s behaviors.

**Conclusions:**

These results underscore the significant influence of parents’ perceptions of social support on their parenting beliefs and behaviors, which may, in turn, affect the development of their children’s mental health. Therefore, parents’ perceptions of social support are likely positively associated with children’s mental health.

## Strengths and limitations

- A notable strength of this study is the employment of a scale with established psychometric soundness to support the validity and reliability of the findings.
- The study has limitations owing to its cross-sectional design, which precludes causal inferences.
- Furthermore, self-report studies are at risk of reporter bias.

## Background

Social support encompasses the provision of material and psychological resources via social networks, facilitating an individual’s capacity to manage stress. It is frequently analyzed owing to its direct and indirect roles in mitigating the adverse effects of various risk factors on physical and psychological health [1]. Individuals perceiving high levels of social support often report enhanced self-esteem, improved self-regulation, better stress management, and increased subjective well-being, which contribute to better mental health outcomes [2]. In this study, social support refers to support from family, loved ones, and friends.

Adolescence is a critical developmental stage marked by significant physical, emotional, and social changes. During this period, individuals begin to assert their independence, which can lead to shifts in their relationships with parents. Adolescents often experience a heightened need for autonomy, which can create tension as they navigate their identity formation and peer influences. Despite these challenges, parental support remains crucial; it provides a foundation for healthy emotional development and coping strategies. Research indicates that positive parental involvement, characterized by open communication and emotional support, can mitigate the risks associated with this tumultuous phase, such as increased susceptibility to anxiety and depression [3, 4]. Understanding the dynamics of the parent–child relationship during adolescence highlights the critical role of parental support in fostering resilience and psychological adjustment in young individuals.

The role of social support in parenting practices is critical, as its lack can lead to feelings of loneliness and neglect, adversely affecting both parents and children. For example, McHale et al. [5] highlights that parents with low levels of perceived social support are more likely to experience increased stress, which in turn can lead to harsher parenting practices and reduced emotional availability for their children. Similarly, Kourkoutas et al. [6] found that single parents often report feelings of isolation, which can result in neglectful behaviors, as they struggle to manage their caregiving responsibilities without adequate support. Furthermore, Bowers et al. [7] demonstrated that parents experiencing social isolation may be less responsive to their children’s needs, leading to negative developmental outcomes. These findings underscore the importance of social support networks in fostering positive parenting practices and mitigating the effects of loneliness and neglect.

Significant research underscores the beneficial impact of social support on family resilience in the face of adversity [8]. Studies have also highlighted the link between social support and effective parenting [9–11]. Social support potentially aids parents by offering insights into child development and effective parenting practices, thereby aligning parental expectations and enhancing parenting skills [12, 13]. Moreover, the elements of social support—such as sources, network size, and satisfaction with support—serve as protective factors in mitigating the stress-related impacts on parenting quality [14, 15].

Conversely, insufficient or inadequate social support is recognized as a risk factor endangering parental mental health, which may result in ineffective parenting practices. Research indicates a positive correlation between social support and constructive mother–child interactions, as well as favorable child development outcomes [16]. Furthermore, more robust social networks are associated with enhanced personal well-being, more positive attitudes, and beneficial impacts on children’s behavior and development [17]. Additionally, social support serves as a buffer against the adverse effects of parenting stress on parental behavior, affecting the frequency of punitive actions, levels of inconsistency, emotional distance, sensitivity, and rejection [18]. In particular, adolescence is marked by rapid biological and psychosocial transformations that significantly influence the dynamics of parent–child relationships. During this period, parents and adolescents must adjust their roles, moving toward a more balanced and egalitarian relationship [19].

Conversely, scant social support can adversely affect families and individuals. Social isolation is also associated with lack of social support, poor mental health and parenting challenges [20]. For example, Families experiencing neglect often report higher levels of social isolation, loneliness, and insufficient support from neighbors, friends, and relatives compared to families without neglect issues [21].

Social support plays a crucial role in helping parents with child-rearing, affecting mothers, fathers, and single parents in unique ways. For mothers, social support alleviates parenting stress, reduces feelings of isolation, and helps improve parenting skills [22]. However, fathers often face barriers to participating in childcare; support from workplace or friends can ease psychological burdens, encouraging more active involvement in parenting [23]. Single parents, who bear the full burden of caregiving, especially benefit from support of friends, family, and community; this reduces isolation and helps manage psychological and emotional challenges [24].

When social support is lacking, mental health issues such as anxiety and depression can worsen, which in turn may diminish the parent’s ability to engage positively with their child(ren) [25–29]. Without support, the psychological strain can lead to inconsistent or imbalanced parenting practices, potentially impacting the child’s well-being. However, with social support, parents are better able to manage stress and maintain a positive approach to parenting, fostering a healthier and more supportive environment for their children.

This study requires a more detailed analysis of the role of social support in parenting. In particular, focusing on the associations between social support and parenting practices for mothers, fathers, and single parents is essential. Research indicates that maternal social support contributes to reducing parenting stress and promoting positive parenting techniques [30]. For fathers, social support has also been confirmed to positively influence parenting behaviors [31]. Additionally, among single parents, a lack of social support exacerbates the relationship between mental health challenges (such as anxiety and depression) and parenting practices [32].

Furthermore, it is important to review the literature on the impact of parents’ perceived social support on children’s psychological adjustment. Studies indicate that when parents experience high levels of perceived social support, their children tend to exhibit better psychological adjustment [33]. Therefore, distinguishing among parenting practices, techniques, and behaviors will enhance our understanding of the role of social support in parenting.

### Study aim

Despite the recognized significance of social support for parenting and child well-being, research focusing on the relationship between parents’ perceived social support and their children’s psychological adjustment remains scarce. This study aims to explore this relationship. The hypothesis of this study posits that the greater the social support available for parents, the lesser their child(ren)’s problem behavior. For instance, increased social support for parents leads to improved emotional regulation in their children [34, 35]. Positive family dynamics are crucial in promoting healthy development.

## Methods

### Participants

This study forms part of a larger research initiative exploring the influence of the parenting environment on children’s social development and adjustment. In 2014, five-year-old children were recruited from 52 kindergartens and 78 preschools in Nagoya, Aichi, a major metropolitan area in Japan. Annual surveys have been conducted since then. The 2022 data collection involved parents of children aged 13–14 years in the eighth grade (N = 1,195) and yielded 602 valid responses. Questionnaires were sent to 1,195 people. Owing to respondents’ inability to send complete survey responses or refusal to answer due to relocation or other reasons, the total number of valid responses was 602. Participants diagnosed with a developmental disability or who failed to complete essential survey items were excluded, resulting in 536 (89.0%) eligible participants. Our research utilized the data collected from the annual surveys initiated in 2014, focusing on children aged 5–14 years. The 2022 survey data were instrumental in assessing how variations in parental social support impact child behavior and mental health.

### Measures

#### Explanatory variable: perceived social support

Perceived social support was assessed using the 12-item Multidimensional Scale of Perceived Social Support (MSPSS), developed by Zimet et al. [36, 37]. This scale employs a seven-point Likert scale, with responses ranging from 1 = strongly disagree to 7 = strongly agree. The MSPSS comprises three subscales, each containing four items, which evaluate perceived support from family, special persons, and friends. Higher scores signify increased levels of social support. The internal consistency for this measure was 0.88.

#### Objective variable: adolescent behavior

Adolescent behavior was evaluated using the Strengths and Difficulties Questionnaire, formulated by Goodman [38]. This 25-item tool is divided into five subscales: emotional symptoms, behavioral problems, hyperactivity, peer relationship issues, and prosocial behaviors. Each subscale includes five questions, offering three response options: 0 = not true, 1 = somewhat true, and 2 = certainly true. The aggregate of the scores from the four problem-oriented subscales (excluding “prosocial behaviors”) generates a total difficulties score, with higher values indicating poorer psychological adjustment. The internal consistency for this measure was 0.86〜0.89.

### Statistical analyses

Multiple regression analysis was utilized to investigate the influence of perceived social support on adolescent behavior, using IBM SPSS Statistics 29.0 for all computations. In all analyses, the variance inflation factor was less than 2.000.

## Results

### Participant characteristics

Table 1 details the socioeconomic characteristics of the participants. The children had an average age of 14.23 years (standard deviation = 0.31), while the average ages for the mothers and fathers were 46.09 years (standard deviation = 4.25) and 47.93 years (standard deviation = 5.31), respectively. We examined the relationship between parental perceived support and their children’s gender and age. We found that children’s gender and age were not related to parental perceived support (Supplemental file).

**Table 1:** Participant attributes

Participant attributes	n	%
*Child’s sex*		
Male	256	47.8
Female	280	52.2
*Family composition*		
Single-parent family	39	7.3
Two-parent family	497	92.7
*Annual household income (million JPY)*		
< 4	86	16.4
4–7	288	54.9
≥ 8	151	28.8
*Maternal educational level*		
Middle or high school	88	16.6
Junior college or vocational school	214	40.4
University or graduate school	228	43.0
*Paternal educational level*		
Middle or high school	123	23.6
Junior college or vocational school	65	12.5
University or graduate school	333	63.9

### Association between social support for parents and children’s mental health

Tables 2, 3, and 4 delineate the relationships between social support for parents and various aspects of children’s mental health. The findings suggest a robust correlation between social support for parents and improved mental health among children, characterized by lower scores for externalizing and internalizing behaviors and higher scores for prosocial behaviors.

**Table 2:** Association between social support for parents and children’s externalizing problemsss

	**B**	**SE**	**β**	***p***	**Adjusted** *R*^**2**^	**VIF**
Social support for parents	-0.110	0.014	-7.671	< .001	0.155	1.051
Child’s sex	-1.128	0.242	-4.670	< .001		1.011
Family composition	0.443	0.577	0.768	0.443		1.070
Annual household income	-0.332	0.196	-1.697	0.090		1.069
Maternal educational level	0.186	0.181	1.029	0.304		1.190
Paternal educational level	-0.261	0.156	-1.674	0.095		1.181

*B* Unstandardized coefficient, *SE* Standard error, *β* Standardized coefficient, *VIF* Variance inflation factor

**Table 3:** Association between social support for parents and children’s internalizing problems

	**B**	**SE**	**β**	***p***	**Adjusted** *R*^**2**^	**VIF**
Social support for parents	-0.182	0.014	-12.682	< .001	0.272	1.050
Child’s sex	0.550	0.242	2.275	0.023		1.011
Family composition	-0.301	0.576	-0.523	0.601		1.070
Annual household income	-0.260	0.196	-1.327	0.185		1.069
Maternal educational level	0.027	0.182	0.150	0.881		1.196
Paternal educational level	-0.043	0.156	-0.276	0.783		1.184

*B* Unstandardized coefficient, *SE* Standard error, *β* Standardized coefficient, *VIF* Variance inflation factor

**Table 4:** Association between social support for parents and children’s prosocial behaviors

	**B**	**SE**	**β**	***p***	**Adjusted** *R*^**2**^	**VIF**
Social support for parents	0.062	0.012	5.276	< .001	0.079	1.051
Child’s sex	0.315	0.198	1.591	0.112		1.014
Family composition	0.256	0.472	0.542	0.588		1.070
Annual household income	0.132	0.160	0.823	0.411		1.068
Maternal educational level	-0.242	0.149	-1.628	0.104		1.196
Paternal educational level	-0.291	0.128	-2.277	0.063		1.186

*B* Unstandardized coefficient, *SE* Standard error, *β* Standardized coefficient, *VIF* Variance inflation factor

Table 2 reveals that children whose parents receive more social support tend to have fewer externalizing problems.

Table 3 reveals that children whose parents receive more social support tend to have fewer internalizing problems.

Table 4 shows that children whose parents receive greater social support tend to exhibit higher levels of prosocial behavior.

## Discussion

This study conducted a comprehensive analysis of the multifaceted relationship between social support provided to parents and the social adjustment of their children. The results revealed a striking pattern: as the level of social support for parents increased, there was a significant decrease in both externalizing and internalizing behavioral problems exhibited by children. Additionally, the findings also indicated that children whose parents reported higher levels of social support displayed enhanced sociality, suggesting that strong parental social networks are instrumental in promoting positive developmental outcomes in children.

Recent research has revealed that a child’s gender plays a significant role in perceived social support. In particular, boys and girls exhibit differences in how they utilize social support and in their recognition of its importance. For example, girls tend to emphasize emotional support, whereas boys are more likely to seek practical assistance [39]. Moreover, the differences in gender at various developmental stages may affect how children acquire and adapt to relevant social support. Changes from early childhood to adolescence can influence how children recognize and utilize social support, ultimately relating to their psychological adjustment [40]. Therefore, the interplay between gender and developmental age is a crucial element in understanding children’s perceptions of social support and psychological development; this highlights the need for further research to consider this perspective.

The concept of social support is crucial in understanding parental well-being [41]. It plays a vital role in alleviating psychological stress through the cultivation and maintenance of social connections, which in turn sustains and/or enhances both mental and physical health [42, 43]. A substantial corpus of research has established that robust social support can render parents more optimistic, which is a key factor in improving their overall mental and physical health, as well as in enhancing effective parenting [44, 45]. When parents perceive themselves as being embedded within strong social networks, their mental health outcomes improve remarkably [46, 47]. This improvement not only influences their emotional well-being, but also has profound implications for their parenting practices and consequently, their children’s development [48].

Social support serves multiple functions that are beneficial to parents. It provides emotional backing, which aids parents in managing their emotional responses to their children’s needs and behaviors. This is particularly critical during challenging parenting situations wherein emotional regulation is essential [49]. Additionally, research suggests that parents who experience substantial social support are more likely to exhibit consistent and positive parenting behaviors. These behaviors are essential for fostering healthy emotional and social development in children [50]. Furthermore, social support equips parents with essential information regarding child development and effective parenting techniques. This access to knowledge not only refines their expectations of child behavior but also enhances their overall parenting skills, leading to better interactions with their children.

Conversely, insufficient social support can jeopardize parental mental health; this, in turn, can result in ineffective parenting practices that adversely impact children’s development. Challenges in parental mental health can lead to diminished positive interactions with young children, increased negative interactions, hostility, impaired communication, and delayed responses to children’s behaviors [51]. The implications of these negative interactions can be profound, contributing to a cycle of poor mental health outcomes for both parents and children. Thus, perceptions of social support among parents may significantly influence their parenting beliefs and behaviors, potentially leading to significant consequences for their children’s mental health development [52].

Moreover, the relationship between social support and parenting is further complicated by cultural and contextual factors. Different cultures may perceive social support in various ways, which can affect how support is provided and received [53]. For instance, in collectivist cultures, social support may be more community-oriented, whereas, in individualistic cultures, support might be more family-focused. This cultural variability highlights the need for a nuanced understanding of how social support impacts parenting and child development across diverse cultural contexts.

In conclusion, the findings of this study underscore the importance of social support in influencing parenting practices and children’s developmental outcomes. Positive perceptions of social support are likely to be associated with better mental health outcomes for children, emphasizing the need for interventions aimed at enhancing parental social networks. Such interventions can potentially mitigate the adverse effects of parenting stress and improve overall family functioning. Therefore, fostering social support systems for parents should be considered a vital component of public health strategies aimed at promoting children’s well-being and mental health.

### Strengths and limitations

A notable strength of this study is the employment of a scale with established psychometric soundness to support the validity and reliability of the findings. Nevertheless, the study has limitations owing to its cross-sectional design, which precludes causal inferences. A longitudinal follow-up of these children and their parents could elucidate the enduring associations between the examined variables.

## Conclusions

This investigation reaffirmed the link between parents’ received social support and children’s social adjustment. Enhanced social support for parents is associated with reduced behavioral and emotional difficulties and increased prosocial behaviors in children.

These findings underscore the critical role of parental perceptions of social support in shaping parenting beliefs and behaviors, which, in turn, can influence the development of children’s mental health. Consequently, these perceptions are likely to be positively correlated with children’s mental health.

## Data Availability

The datasets generated and/or analyzed during the current study are available from the corresponding author upon reasonable request.

## Abbreviations

MSPSS: Multidimensional Scale of Perceived Social Support
SE: Standard error
